# A Rare Case of Clopidogrel-Induced Migratory Polyarthritis in a Patient With Recent Myocardial Infarction

**DOI:** 10.7759/cureus.22042

**Published:** 2022-02-09

**Authors:** Zahid Khan, Yousif Yousif, Mohammed Mohammed, Ayub Khan, Abdullah Afghan, Syed Aun Muhammad, Vinod Warrier, Animesh Gupta

**Affiliations:** 1 Cardiology, Royal Free Hospital, London, GBR; 2 Internal Medicine, Barking, Havering and Redbridge University Hospitals NHS Trust, London, GBR; 3 Emergency Medicine, Barking, Havering and Redbridge University Hospitals NHS Trust, London, GBR; 4 Internal Medicine, Daisy Hill Acute Hospital, Newry City, Dublin, IRL; 5 Cardiology, Mid and South Essex NHS Foundation Trust, Southend on Sea, GBR; 6 Internal Medicine, Mid and South Essex NHS Foundation Trust, Southend on Sea, GBR; 7 Acute Internal Medicine, Barking, Havering and Redbridge University Hospitals NHS Trust, London, GBR

**Keywords:** degenerative joint disease, antiplatelet therapy, acute pain, clopidogrel hypersensitivity, gout disease, migratory arthralgia, st-elevation myocardial infarction (stemi), drug-related side effects and adverse reactions

## Abstract

Clopidogrel is an antiplatelet drug used for secondary prevention of myocardial infarction, and it is also used in patients with cerebrovascular ischemia. Patients with acute myocardial infarction tend to be on dual antiplatelet therapy for 12 months followed by aspirin lifelong to prevent the risk of stent thrombosis. The most common side effects of clopidogrel are bleeding, neutropenia, and rash; however, arthritis is also one of the rare side effects. We present a case of a 53-year-old patient who had a recent myocardial infarction and was commenced on dual antiplatelet therapy in the form of aspirin and clopidogrel. He started to have severe joint pain, particularly in his knees and shoulders, and was not able to mobilize anymore only three weeks after starting the medications. His clopidogrel was stopped and the patient showed dramatic improvement within three to four days after discontinuation with complete resolution one week later.

## Introduction

Clopidogrel is an antiplatelet drug belonging to the class of thienopyridines, and it is a novel adenosine diphosphate (ADP) selective agent with very high antiplatelet aggregating activity [1]. Clopidogrel prevents platelet aggregation by inhibiting the binding of ADP to its platelet-low affinity receptors and sparing the high-affinity binding sites [1]. Dual antiplatelet therapy (DAPT) for 12 months is the cornerstone of acute myocardial infarction (AMI) in patients with primary percutaneous coronary intervention (PPCI). The combination therapy can be different across centres and some cardiac centres use other drugs like ticagrelor or prasugrel with aspirin in patients with AMI. The active metabolite form of the thienopyridines group of drugs irreversibly inhibits the ADP binding P2Y12 receptor on the platelet membrane surface [2]. This interaction between ADP and P2Y12 plays an important role in platelet aggregation and the prevention of plaque formation [3].

Studies have shown that dual-platelet therapy after AMI reduces the risk of stent thrombosis, further myocardial infarction, and cardiovascular death, and DAPT has been in use following AMI for a few decades now [4]. Clopidogrel is a relatively safe drug and the Clopidogrel versus Aspirin in Patients at Risk of Ischemic Events (CAPRIE) trial demonstrated the superiority of clopidogrel over aspirin in patients with atherosclerotic vascular disease to reduce the risk of stroke and myocardial infarction in patients with a similar safety profile [5]. There was no difference in the safety profile data for both aspirin and clopidogrel in this cohort of patients and the most common side effects reported with both aspirin and clopidogrel included rash, diarrhoea, upper gastrointestinal discomfort, and intracranial and gastrointestinal haemorrhages respectively [5]. Similarly, another trial te “Clopidogrel in Unstable Angina to Prevent Recurrent Ischemic Events” (CURE) trial showed a significant benefit of aspirin and clopidogrel combination therapy in patients with unstable angina and myocardial infarction compared to aspirin alone [6]. The most common side effects noted with clopidogrel in this study were bleeding risk, gastrointestinal problems, back pain and arthralgia, and these side effects were more prominent when it was used in combination with aspirin.

## Case presentation

We present a case of a 53-year-old patient with a past medical history of hypertension, angina, high body mass index (BMI), hypercholesterolaemia, gout and type 2 diabetes mellitus, who was also a smoker. He presented to our hospital with non-ST elevated myocardial infarction (NSTEMI), had primary percutaneous intervention (PPCI) to the right coronary artery (RCA) three weeks prior and was commenced on DAPT for 12 months. His medications included metformin 1 gram (gm) twice daily (BD), ramipril 2.5 mg once daily (OD), aspirin 75 mg OD, clopidogrel 75 mg OD, bisoprolol 2.5 mg OD, atorvastatin 80 mg omni nocte (ON), lansoprazole 20 mg OD and glyceryl trinitrate spray (GTN) as required. He had been started on clopidogrel and bisoprolol after the AMI recently.

He had presented to the acute medical unit (AMU) three weeks after his myocardial infarction (MI) with severe arthralgia and inability to mobilize. The clinical examination was unremarkable, and there was no obvious joint swelling or redness. His arthralgia was predominantly in his shoulder, back, knees and hips, and he was unable to get up from a sitting position due to significant joint stiffness. His lab results were unremarkable apart from mildly elevated white cell count and neutrophils, and c-reactive protein (CRP) was 25 mg/l (normal value 5 mg/l). His uric acid level (5.4 mg/dL) and erythrocyte sedimentation rate (ESR) level (24 mm) in the first hour were normal. He was apyrexial and his joint mobility was very limited due to stiffness. His symptoms started three to four days after starting clopidogrel. There was no evidence of gouty tophi on clinical examination. Radiographs of his knees and hips showed only minor degenerative changes that could not explain his symptoms. His autoimmune screen, myeloma screen and Lyme disease and Brucella screening were all negative. He did not complain of any muscle ache and his creatinine kinase level was normal. His urine dip was negative, and he did not have a rash.

He was commenced on ticagrelor 90 mg BD after receiving a loading dose of 180 mg, and his clopidogrel was stopped. The patient showed significant improvement in his symptoms within three to four days of stopping his clopidogrel, and his symptoms showed complete resolution within a week. He received paracetamol and codeine for pain relief, which he also discontinued after one week of treatment.

## Discussion

Clopidogrel-induced arthritis is rare, nevertheless, there are published case reports of clopidogrel-induced migratory polyarthritis [6-8]. Clopidogrel-induced arthritis is a diagnosis of exclusion by excluding other causes of migratory polyarthritis such as autoimmune arthritis, infectious arthritis, reactive arthritis, gout, pseudogout and serum sickness disease. This patient had a negative autoimmune screen, and his inflammatory markers were normal. In addition, his arthralgia resolved with the cessation of clopidogrel.

Another study reported a 76-year-old patient to have developed widespread pruritus without any rash and painful swollen metacarpophalangeal joints two weeks after commencing clopidogrel [9]. She had red and hot joints with evidence of synovitis in metacarpophalangeal joints. She had an elevated CRP of 81 mg/l and ESR 86 mm in the first hour. She had a normal uric acid level and radiographs showed soft tissue swelling and her clopidogrel was stopped. She improved both clinically and biochemically a week after the cessation of clopidogrel. In comparison, our patient had a normal CRP and ESR, and his joint pain improved four days after his clopidogrel was stopped, showing full recovery after a week.

Another case reports a 63-year-old man who was an ex-smoker, had type 2 diabetes mellitus and had an uncomplicated subarachnoid haemorrhage in 1984. He was commenced on clopidogrel alongside his regular medications lisinopril and diltiazem after undergoing coronary artery bypass surgery. He presented to his doctor three weeks later with severe pain in his right knee, which was tender, and he was unable to weight bear. His urate concentration was normal and his ESR was mildly elevated at 47 mm in the first hour. His symptoms completely resolved after discontinuation of clopidogrel [9].

Most cases of clopidogrel-induced polyarthritis are on maintenance doses and only a single case report is based on a loading dose of clopidogrel [7]. Cheema et al., based on their study of 62 patients, reported that patients develop clopidogrel hypersensitivity, such as fever and arthralgia, after a median time of five days [10] and our patient symptoms also started three to four days after the initiation of clopidogrel.

## Conclusions

In conclusion, clopidogrel-induced arthritis is rare; however, clinicians need to be aware of this and should always consider it as a possible differential in patients presenting with acute arthralgia after an acute myocardial infarction when they are commenced on clopidogrel. The inflammatory markers may vary from being normal to having mild elevation. Clinical examination is of fundamental importance and these patients respond to the cessation of the medication. It is also important to remember that clopidogrel-induced arthritis is a diagnosis of exclusion and other causes should be excluded first.
